# Gamma‐secretase activity, clinical features, and biomarkers of autosomal dominant Alzheimer's disease

**DOI:** 10.1002/alz70856_098877

**Published:** 2025-12-24

**Authors:** Stephanie A. Schultz, Lei Liu, Hyun‐Sik Yang, Dennis J. Selkoe, Jasmeer P. Chhatwal

**Affiliations:** ^1^ Massachusetts General Hospital, Brigham and Women's Hospital, Harvard Medical School, Boston, MA, USA; ^2^ Ann Romney Center for Neurologic Diseases, Department of Neurology, Brigham and Women's Hospital, Harvard Medical School, Shiga, Japan; ^3^ Department of Neurology, Brigham and Women's Hospital, Boston, MA, USA; ^4^ Massachusetts General Hospital, Boston, MA, USA; ^5^ Department of Neurology, Brigham and Women's Hospital, Harvard Medical School, Boston, MA, USA; ^6^ Brigham and Women's Hospital, Harvard Medical School, Boston, MA, USA; ^7^ Massachusetts General Hospital, Harvard Medical School, Boston, MA, USA

## Abstract

**Background:**

Rare cases of autosomal dominant Alzheimer's disease (ADAD) can provide unique insights into Alzheimer's disease (AD) pathobiology. Pathogenic variants in *PSEN1* or *PSEN2* are the most common causes of ADAD. PSEN1 or PSEN2 can form the catalytic core of the γ‐secretase complex, which, in turn, directly mediates the production of longer, aggregation‐prone Aβ peptides relative to shorter, non‐aggregating peptides. Given the great deal of heterogeneity seen in ADAD ‐ with ages of symptom onset (AAO) ranging from the 20s to 70s – we examined whether variant‐level variations in γ‐secretase function and Aβ production can explain variant‐level differences in ADAD progression.

**Method:**

Expression of mutant or wild‐type PSEN1/2 in cell culture and measurement of Aβ production, coupled with literature‐derived AAO and *in vivo* clinical, cognitive, and biomarker data from the Dominantly Inherited Alzheimer's Network Observational Study (DIAN).

**Result:**

Aβ peptide production from 161 known pathogenic mutations in *PSEN1* and 70 variants in *PSEN2* were assessed *in vitro*. Aβ production (particularly the Aβ42/40 and Aβ37/42 ratios) by individual *PSEN1* variants was strongly correlated with AAO, with more abnormal γ‐secretase function associated with earlier AAO. Aβ production among *PSEN2* variants with known homologs in *PSEN1* showed strong associations with AAO, whereas Aβ production in *PSEN2* variants without known *PSEN1* homologs was similar to wild‐type for many (but not all) variants. In a subset of 55 *PSEN1* variants with available data from DIAN, variant‐level Aβ production profiles correlated with all clinical (CDR), cognitive (Logical Memory; MMSE), and biomarker (Amyloid PET, structural MRI, CSF ptau) measures examined, as well as with observed longitudinal rates of biomarker and clinical progression of ADAD. Further, accounting for variant‐level Aβ production measured *in vitro* improved prediction of *in vivo* clinical, cognitive, and biomarker trajectories beyond history derived estimates of AAO.

**Conclusion:**

These data and the associated literature strongly suggest that the course of ADAD is fundamentally shaped by variant‐level effects on γ‐secretase function and Aβ production. In addition to providing a unique line of support for the “amyloid hypothesis” of AD, these results provide compelling support for γ‐secretase modulation (GSM) as a promising approach to treat AD.

